# Tackling Dysfunction of Mitochondrial Bioenergetics in the Brain

**DOI:** 10.3390/ijms22158325

**Published:** 2021-08-03

**Authors:** Paola Zanfardino, Stefano Doccini, Filippo M. Santorelli, Vittoria Petruzzella

**Affiliations:** 1 Department of Medical Basic Sciences, Neurosciences and Sense Organs, University of Bari Aldo Moro, 70124 Bari, Italy; paola.zanfardino@uniba.it; 2 IRCCS Fondazione Stella Maris, Calambrone, 56128 Pisa, Italy; stefano.doccini@fsm.unipi.it

**Keywords:** mitochondria, mitochondrial DNA, nervous tissue, OxPhos complexes, bioenergetics, genomics, proteomics, mitochondrial diseases

## Abstract

Oxidative phosphorylation (OxPhos) is the basic function of mitochondria, although the landscape of mitochondrial functions is continuously growing to include more aspects of cellular homeostasis. Thanks to the application of -*omics* technologies to the study of the OxPhos system, novel features emerge from the cataloging of novel proteins as mitochondrial thus adding details to the mitochondrial proteome and defining novel metabolic cellular interrelations, especially in the human brain. We focussed on the diversity of bioenergetics demand and different aspects of mitochondrial structure, functions, and dysfunction in the brain. Definition such as ‘*mitoexome*’, ‘*mitoproteome*’ and ‘*mitointeractome*’ have entered the field of ‘mitochondrial medicine’. In this context, we reviewed several genetic defects that hamper the last step of aerobic metabolism, mostly involving the nervous tissue as one of the most prominent energy-dependent tissues and, as consequence, as a primary target of mitochondrial dysfunction. The dual genetic origin of the OxPhos complexes is one of the reasons for the complexity of the genotype-phenotype correlation when facing human diseases associated with mitochondrial defects. Such complexity clinically manifests with extremely heterogeneous symptoms, ranging from organ-specific to multisystemic dysfunction with different clinical courses. Finally, we briefly discuss the future directions of the multi-omics study of human brain disorders.

## 1. Introduction

The panoply of mitochondrial functions reflects on highly heterogeneous clinical presentations when an error in a mitochondrial protein or function occurs. Mitochondria are dynamic and mobile organelles representing a hub where exchange of information among the nucleus and other cellular compartments takes place to modulate energy production and metabolites provision to the cell’s specific needs and nutrient availability [1]. The basic function of mitochondria is the generation of more than 90% of cellular energy via the oxidative phosphorylation (OxPhos) system [2] but, in addition, they play many roles in the different types of cells: compartmentalize metabolites for the maintenance of redox homeostasis; function as centers for metabolic waste management [3]; surveil calcium homeostasis [4]; initiate caspase-dependent apoptosis and other intermediate cellular stress response [5]; provide sulfur metabolism and iron-sulfur cluster biogenesis [6,7]; house the synthesis of cardiolipin, steroids, quinone, and heme [8,9]; breakdown fatty acids through β-oxidation; and serve as a metabolic platform for the tricarboxylic acid (TCA), and urea cycles [10]. All these functions include homeostatic regulation of organelle morphology and dynamics [11], quality control [12], and participation in the immune response [13,14]. Alteration of each of the above functions and activities can have different effects according to the specificity of the organ and cell type, but alteration of mitochondrial energy production can impact tissues with the highest energy requirements such as the nervous system, both central (CNS) and peripheral (PNS) [15,16]. 

The term ‘mitochondrial medicine’ categorizes the ample array of clinical presentations associated with all types of mitochondrial defects having directly or secondarily defect of one or several mitochondrial functions although ‘mitochondrial diseases’ traditionally indicate dysfunction of the OxPhos system [6,17]. The direct link between human disease and the genetic alteration of a mitochondrial function has found a breakthrough with the application of -*omics* technologies (i.e., genomics, transcriptomics, proteomics, metabolomics, and epigenomics, etc.). Rapidly, high-throughput *omics* techniques—that is detection of biologically significant differences, even if not high magnitude changes, in a multitude of molecular constituents in organisms supported by sophisticated bioinformatics tools—have allowed progress in cataloging the predicted human mitochondrial proteins thus revealing new details and providing clues to elucidating still unknown basic aspects of mitochondrial structure and function. These novel high-throughput techniques have enhanced the final diagnosis of several mitochondrial disorders. This is a very relevant aspect, especially considering that mitochondrial diseases individually are rare but are probably the most frequent genetic disorder in adults (incidence of 1 in 5000 live births) [18]. More recently, genome editing technology applied to neural cultures and cerebral organoids generated from patients-derived iPSCs is revolutionizing the landscape and offering new opportunities for understanding the pathogenetic effects of mutations in nervous tissue. 

This review aims to focus on the dysfunction of OxPhos defects mostly in the nervous system to highlighting the contributions of powerful omics technologies to mitochondrial medicine to land from the laboratory to the clinic.

## 2. Mitoexome, Mitochondrial Proteome, and Mitointeractome

**Figure 1:**
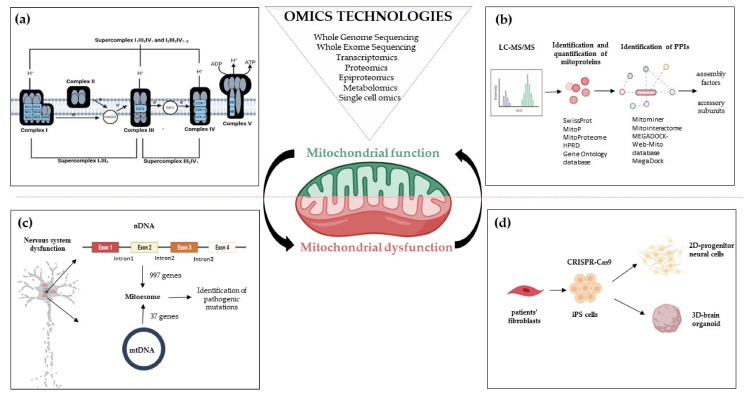
Omics strategies advance in understanding mitochondrial function and dysfunction in brain disorders related to OxPhos gene mutations. Mitochondrial bioenergetics involves activities whose function and structure have been deeply elucidated by *omics* technologies; (**a**) The introduction of high-resolution technologies has been resolutive to deepen the structure of the respiratory chain complexes and supercomplexes; (**b**) Quantitative proteomics, e.g., LC-MS/MS enable the identification and quantification of mitoproteins and provide large amounts of data. Through Network-based approaches analyzing protein-protein interactions, the huge amount of information allows the discovery of novel accessory subunits and assembly factors of the five multi-subunit enzyme complexes; (**c**) The re-sequencing carried out with MitoExome increases the possibility of identifying new or previously reported mutations in both mitochondrial and nuclear genes in patients; (**d**) Novel multi-omics analysis, based on single-cell *omics*, is applied to two-dimensional (2D) neural cultures and three-dimensional (3D) cerebral organoids generated from patients-derived iPSCs that can be engineered by CRISPR/Cas9. Abbreviations: LC-MS/MS: Liquid Chromatography with tandem mass spectrometry; PPIs: Protein-protein Interactions; nDNA: nuclear DNA; mtDNA: mitochondrial DNA; iPS cells: Induced Pluripotent Stem cells.

Before Next-Generation Sequencing (NGS) improved our understanding of how mutations cause diseases, first attempts to identify the mitochondrial proteome were based on ‘*cyberscreening*’ of available genome databases. This allowed the discovery of few human mitochondrial genes presenting orthologs in lower eukaryotes. An example of the cyberscreening strategy used *Saccharomyces cerevisiae* proteins as ‘probes’ to identify *BCS1*, *PET112*, *SCO1*, *COX15*, and *COX11*, five yeast genes that present orthologs (respectively, *BCS1L*, *GATB*, *SCO1*, *COX15*, and *COX11*) in humans [19]. Except for *COX11*, a COX-assembly, all genes have been implicated in mitochondrial diseases [OMIM 603647.0001-603647.0013; OMIM 603645.0001-603645.0002; OMIM 603644.0001-603644.0002; OMIM 603646.0001-603646.0004], see paragraphs 4.3 and 4.4. To date, whole-exome (WES) and whole-genome (WGS) resequencing have dramatically enhanced the ability to identify the underlying gene mutations in patients with isolated or multiple mitochondrial respiratory chain complex defects [20,21]. The collection of mt genes and coding exons of the 1034 nuclear genes encoding the human mitochondrial proteome is defined as ‘MitoExome’ [22,23]. This multigene panel is useful in performing targeted resequencing of the OxPhos nuclear genes because it includes not only the 77 nuclear structural OxPhos subunits and the 37 mitochondrial (mt) DNA genes including the 13 structural genes for OxPhos subunits [24] but also genes for mitochondrial proteins either already known or not to be associated with a specific mitochondrial disease, including assembly factors and electron carriers’ genes which represent a large fraction of the overall mitochondrial genes that can cause mitochondrial dysfunction [21]. Application of *MitoExome* resequencing provides novel mutation candidates, enables the discovery of unusual clinical variants [25,26] and new clinical phenotypes [26] (Figure 1). Furthermore, the integration of MitoExome sequencing with the study of mitochondrial proteome potentiates the detection of variants causing protein destabilization and/or aberrantly low expression [27].

**Figure 2:**
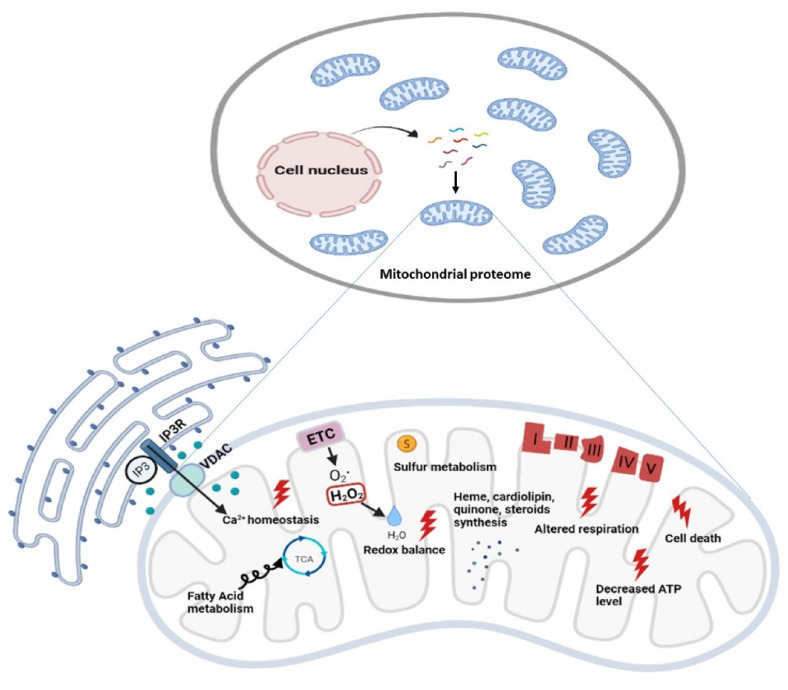
Functional diversity of mitochondrial proteins and bioenergetics consequences of OxPhos system dysfunction. The mammalian mitochondrial proteome includes both mitochondrial and nuclear DNA- encoded proteins. Most of the proteins required for the various activities in which mitochondria are involved are encoded by the nuclear genome, whereas the mitochondrial energy-producing system, i.e., the OxPhos complexes, has either mitochondrial DNA (mtDNA) and nuclear DNA (nDNA) encoded components. The enlarged mitochondrion shows most of the bioenergetics consequences (indicated by the red bolt lightning) of genetic defects involving the OxPhos complexes. Abbreviations: IP3: Inositol Trisphosphate; IP3R: Inositol Trisphosphate Receptor; VDAC: Voltage-dependent anion channel; ETC: Electron Transport Chain; TCA: Tricarboxylic Acid Cycle; ATP: Adenosine triphosphate.

Biochemical and ultrastructural characterizations have uncovered the heterogeneity of mitochondria in their function, trafficking patterns, lifespan, and morphology across cell types and different cellular compartments. Different tissues, cell types, and cellular states have unique signatures of protein localization to mitochondria. In the proteomic comparison of the mitochondrial proteins, almost half are found as *core* components in virtually all tissues, whereas the remaining are tissue-specific [28,29]. The study of mitochondrial proteome starts with the isolation of mt compartment from cells and tissues and stands behind the availability of methodologies to isolate pure mitochondria from different sources to define exactly the function of each protein in each cell type of the human body [30]. The performance of proteomics analysis is driven by the reduction of sample complexity, enhancement of mass spectrometry (MS) power of resolution, and the possibility to reduce the contamination of the sample with non-mitochondrial proteins owed to chemical and physical similarities between mitochondria and other cellular components (e.g., lysosomes). Since the initial rough estimates, it has been suggested that the mammalian mitochondrial proteome encompassed about 1000–1500 distinct proteins—including the 13 mtDNA-encoded proteins [24]—that represent an important subset of the ~20,000 distinct mammalian proteins [31,32] (Figure 2). 

Quantitative two-dimensional (2D) gels of highly purified mitochondria estimated ~1500 distinct spots [33], a number higher than the ~1000 distinct protein products encoded by the genomes of alpha-proteobacteria, which are the closest living relatives of modern-day mitochondria [34]. Several databases have been used to integrate the experimental data with bioinformatic predictions based on mitochondrial localization or interaction. For example, the MitoProteome is an object-related database developed at the UCSD Supercomputer Center, which contains information on mitochondria-localized proteins [35,36]. Each entry in the MitoProteome corresponds to a gene encoding a protein that is localized within mitochondria and its basic information, along with annotations of isoforms, splice variants, and functions of the corresponding protein. To date, the most comprehensive study elucidating the mitochondrial proteome of different mammalian tissues is represented by the MitoCarta inventory [29,37]. This catalog combines multiple experimental and computational approaches, i.e., mass spectrometry (MS) analysis of mitochondria isolated from 14 mouse tissues, large-scale GFP-fusion microscopy analysis, and bioinformatics using data mining, prediction, evolutionary conservation, and a Bayesian integration of seven additional data sources. The first release was represented by MitoCarta1.0 (http://www.broadinstitute.org/pubs/MitoCarta/; accessed 25 July 2021) which contained about 1000 distinct gene *loci* [29]. Updated in 2016, MitoCarta 2.0 listed about 1200 genes [37]. Another dedicated database that collected, curated, and annotated information on mitochondrial proteins is the *MitoMiner database* (http://mitominer.mrc-mbu.cam.ac.uk/; accessed 25 July 2021) [38] (version 4.0, 2018). It is based on the literature and proteomics data based on both LC-MS and 2D gel studies, antibody staining, and other subcellular localization data, and provides a collective score for each protein’s probability to have the mitochondrial association. MitoMiner records mitochondrial proteins from 12 organisms [38]. Using the data contained within MitoMiner, the *Integrated Mitochondrial Protein Index* (IMPI) was also developed (http://www.mrc-mbu.cam.ac.uk/impi; accessed 25 July 2021). IMPI version Q2 (2018) contains 1626 human genes that encode mitochondrially localized proteins, 1184 known to be mitochondrial and 442 predicted to be mitochondrial. The large amount of information provided by mito-databases as MitoMiner 4.0 v2018 JUN (http://mitominer.mrc-mbu.cam.ac.uk; accessed 25 July 2021), makes it possible to define different score systems for mitochondrial confidence combining data from various mitochondrial and functional annotation databases. These strategies allow increasing the stringency of protein accepted as inherently mitochondrial [39]. An exhaustive list of the major data sources loaded with the latest version and links to the relevant resources is reported in the Data Sources section of the Mitominer (https://mitominer.mrc-mbu.cam.ac.uk/release-4.0/dataCategories.do; accessed 25 July 2021). 

More recent advances in the experimental proteomic approaches, specifically in labeling and MS methods, have further expanded and defined the known mitochondrial proteome and have simultaneously revealed the sub-mitochondrial localization of many of them [40,41]. A novel spatial proteomics pipeline demonstrated that many proteins cannot be classified to a single localization as they either transit between compartments or carry out their functional role(s) in multiple locations [41]. The redundant functions, or functions affecting multiple cellular processes, rendered difficult the study and it was estimated that about ~20% of mitochondrial proteins remained uncharacterized [42]. 

Along with technological progress that has enabled the discovery of approximately 78,120 human proteins [based on The UniProt Knowledgebase (UniProtKB), as of 23 February 2021], derives the challenge of identifying a large amount of potential protein-protein interactions (PPIs). An example of the network-based approaches analyzing protein-protein interaction is represented by MitoInteractome, a web-based portal containing 6549 protein sequences extracted from SwissProt (http://www.expasy.ch/sprot/; accessed 25 July 2021), MitoP (http://www.mitop.de:8080/mitop2/; accessed 25 July 2021), MitoProteome (http://www.mitoproteome.org/; accessed 25 July 2021), HPRD (http://www.hprd.org; accessed 25 July 2021) and Gene Ontology database (http://www.geneontology.org; accessed 25 July 2021). This enables the elucidation of integrative mitochondrial functions and can expedite the discovery of novel interactions which otherwise may have been missed using traditional experimental techniques. MEGADOCK [43,44], a structure-based PPI prediction method, was first developed and then followed the MEGADOCK-Web-Mito database which is a PPI prediction data archive, that includes prediction results for protein pairs of 654 mitochondria-related human proteins [45]. All these approaches have been key in the study of PPI as a means to infer functions for uncharacterized proteins and to enable the discovery of novel proteins, e.g., several complex I assembly factors [46,47] (Figure 1). 

For expert reviews on the details about the technical approaches, the required bioinformatics pipelines, and how (multi)omics technologies can help in studying the dysfunction of mitochondrial bioenergetics, see [48,49]. 

## 3. Diversity of Bioenergetics Demand in the Brain

The brain relies on glucose metabolism for ATP generation and many other activities and an inappropriate supply of either glucose or oxygen degrades brain function. The principal energy request of the brain is due to activities of the neuronal signaling that include resting and action potentials, glutamate cycling, post-synaptic Ca2, postsynaptic receptors, while the activities of the non-signaling, e.g., turnover of proteins, phospholipids, and nucleic acids, remodeling of the actin in the cytoskeleton, axonal transport, mitochondrial proton leak, etc., are less demanding. Specifically, gray matter and white matter have different energetic requests for non-signaling (30% versus 80%, respectively) and signaling (70% versus 20%, respectively) activities [50,51]. These findings would suggest that the energy demands of signaling activities in gray matter are mainly due to synaptic activity while the energetic demands in white matter satisfy the request of billions of unmyelinated axons and glial cells [50]. Beyond ATP generation, glucose is important for the synthesis of several molecules within the brain, including neurotransmitters and neuromodulators. For these reasons, mitochondria are quite heterogeneous as anatomical localization, activity, and metabolism at regional, cellular, subcellular levels and during differentiation, when the upregulation of mitochondrial metabolism is the basis of cell proliferation in neuronal stem cells and progenitor cells. Although different regions of the brain contain about half as many mitochondria as the heart, the mitochondria of the brain are qualitatively different to support the high metabolic demand that requires, for example, close cooperation between neurons and astrocytes [52]. Astrocytes are metabolically and structurally supportive [52,53,54] and are crucial in neurotransmission [55,56] and behavior [57,58]. The ATP utilized by neurons is produced by the OxPhos process, while most of the energy needs of astrocytes are met by glycolysis [59,60]. The mitochondrial ATP production per molecule of glucose oxidized is ~16 times more than glycolysis. The survival of neurons requires OxPhos [52] and in mature neurons, the local ATP supply provided by mitochondria is used to regulate axonal and dendritic development, axonal regeneration, as well as contributing to synaptic transmission and plasticity. The different energy metabolisms of the two cell types are closely coupled, with astrocytes releasing the glycolytic end-product, lactate, which is used by neighboring neurons to drive OxPhos [61,62]. 

An example of heterogeneity of mitochondria in metabolic enzyme diversity has been provided by a study comparing the mitochondrial proteome of the three major cerebellar cell types: Granule cells (GC), the most abundant excitatory neuron; Purkinje cells (PC), the major inhibitory neuron of the cerebellum and astrocytes [63]. In the adult cerebellum, ~15% of the annotated mitochondrial proteome was shown to be differentially regulated among the three cell types. Fatty acids were more efficiently metabolized by astrocytic than neuronal mitochondria due to the enrichment of two beta-oxidation enzymes, i.e., short-chain-specific acyl-coenzyme A dehydrogenase and carnitine palmitoyl-transferase 1a, an enzyme that limits the rate of oxidative reactions of long-chain fatty acids [63]. In particular, the mitochondrial proteome of astrocytes showed a remarkable enrichment of peroxisomal proteins, some of which are known to have a double localization (i.e., catalase) [64] or binding to mitochondria (i.e., Eci2 and Pex11b).

In the same work, the mitochondrial calcium uniporter (MCU) [4,63,65] and its regulators were detected mostly in GC [63]. Recent studies suggest that the markedly different modes of ATP production in the neurons and astrocytes reside also in the supra-organization of the mitochondrial respiratory chain in supercomplexes (see Section 4.6 paragraph) able to regulate different rates of respiration and mitochondrial ROS production [66].

**Figure 3:**
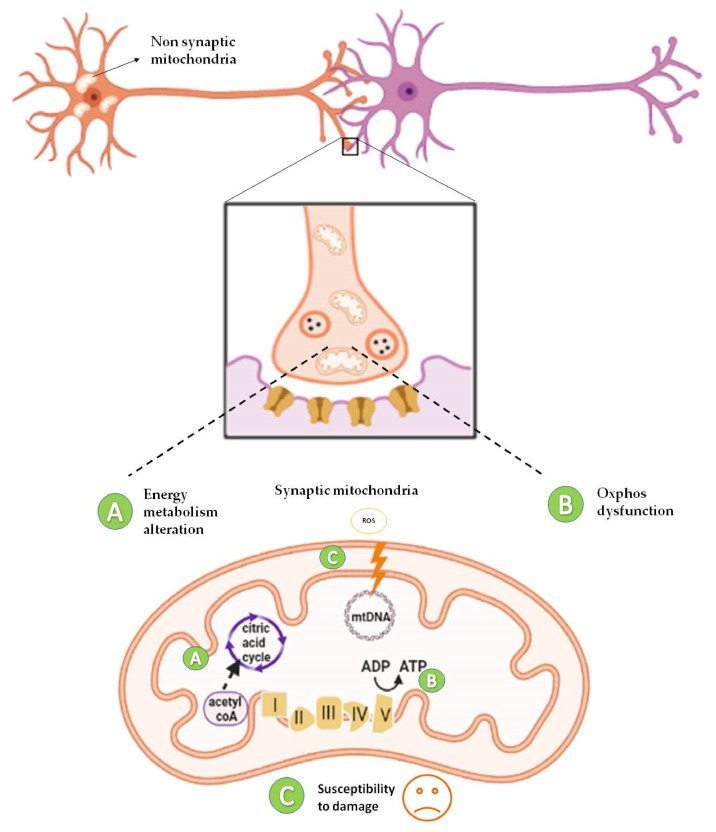
Diversity of mitochondrial proteome in synaptic and non-synaptic mitochondria. Synaptic mitochondria show defects in energy metabolism due to low levels of Pyruvate dehydrogenase (PDH) subunits [68] (**A**), reduced expression of complex I [67,68], II, and IV [68] (**B**), and increased susceptibility to damage (increased mtDNA deletions) [68] (**C**), compared to non-synaptic mitochondria. ADP: Adenosine diphosphate; ATP: Adenosine triphosphate.

The brain mitochondrial proteome is not a *unicum* also when considering synaptic and non-synaptic mitochondria (sMito and nsMito). Proteomic profiling of sMito vs. nsMito revealed mitochondrial complex I as an upstream regulator of degenerative processes associated with a high range of age-related neuropathologies characterized by synaptic dysfunction [67]. In a separate study, an accurate analysis of quantitative proteomics was performed to differentiate sMito and nsMito using Stable Isotope Labeling with Aminoacids in Cell culture (SILAC) labeled mitochondria from cultured cells as an internal standard. In SILAC, cells are differentially labeled by growing them in a ‘light’ medium, containing normal amino acids, or a ‘heavy’ medium, containing a stable isotope [68]. Significant differential expression was shown for 522 proteins involved in several pathways including the OxPhos system, mitochondrial fission/fusion, calcium transport, and mtDNA replication and maintenance. Lower levels of Pyruvate dehydrogenase (PDH) subunits in the synapse to other parts of the cell and reduced expression of complex I, II, and IV (expect for COX4I2) suggested decreased bioenergetic function of sMito compared to nsMito [68]. Consistent with this finding, sMito exhibited increased age-associated mtDNA deletions and reduced levels of TFAM and mtSod2, suggesting a reduced ability of sMito to withstand ROS, thus providing insights into synaptic mitochondrial susceptibility to damage [68] (Figure 3). 

The extreme heterogeneity of mitochondria activities and functioning has been recently shown by a novel and fine imaging approach that specifically allows to label and monitor mitochondrial translation products for microscopic fluorescent imaging. In neuronal cultures, mitochondrial translation was monitored in axonal and dendritic mitochondria as well as in pre-and post-synaptic regions of neurites by specifically labeling the peptides newly synthesized by mitochondrial ribosomes, revealing that not all mitochondria translate to the same extent in different cell types [69]. Finally, the fundamental role of mitochondria during neurogenesis has been recapitulated in the cellular and organoid model of Leigh syndrome (LS), a severe manifestation of mitochondrial disease in children [70]. Mutations in *SURF*1, a complex IV assembly gene, cause neuronal impairment because of defective metabolic programming of neural progenitor cells (NPCs) that prevents the establishment of neuronal morphogenesis. Using CRISPR/Cas9 engineered *SURF*1 patient-derived iPSCs, a human model of LS was developed. Single-cell RNA-sequencing and multi-omics analysis revealed compromised neuronal morphogenesis in mutant 2D neural cultures and 3D brain organoids (Figure 1d). The defects already emerged at the level of NPCs, which were unable to shift toward OxPhos and retained a proliferative glycolytic state that fails to instruct neuronal morphogenesis. Interestingly, gene augmentation and *PGC1A* induction via Bezafibrate treatment inducing mitochondria biogenesis supported the metabolic programming of LS NPCs, leading to restored neuronal morphogenesis [70]. It is interesting to point out that the current understanding of LS is that the disease is caused by neuronal degeneration. This interpretation had led to experimental treatment schemes focused on antioxidants to prevent the build-up of damaging free radicals. The multi-omics analysis in 2D and 3D models adopted by Prigione [70] provided a novel perspective to LS pathology by showing that the disease mechanisms may not necessarily involve a redox imbalance but rather an impairment of neuronal morphogenesis following the loss of NPC commitment. Evidence that Surf1 impairment may affect the neurogenesis was described also in the *SURF*1-knock out swine model that shows a disorganized cortical structure with several immature neurons and developing of a severe early-onset neurological phenotype [71]. These findings overall suggest that mutations associated with mitochondrial diseases could impair neurogenesis and shift the view of therapeutic approaches that might lead to novel interventions aiming at promoting the reestablishment of physiological neurogenesis [72] rather than merely preventing the degeneration of mature neurons.

## 4. Structure, Assembly, and Disorders of Bioenergetics Complexes

The development of mito-omics-based approaches has been crucial in understanding the functional and bioenergetic consequences of mutations responsible for the onset of primary mitochondrial diseases. The OxPhos is the enzymatic machinery by which mitochondria produce the ATP needed by the cells. The reactions are performed by five multimeric enzyme complexes (EC): Complex I (EC 1.6.5.3) or NADH-Ubiquinone Reductase, CI, 45 subunits; Complex II (EC 1.3.5.1) or Succinate-Ubiquinone Oxidoreductase, CII, 4 subunits; Complex III (EC 1.10.2.2) or Ubiquinol: cytochrome c (cyt c) oxidoreductase, CIII, 10 subunits; Complex IV (EC 1.9.31) or Cyt *c* oxidase (COX), CIV, 13 subunits; Complex V (EC 3.6.14) or ATP synthase, CV, 16 subunits; and two-electron transport carriers, namely, ubiquinone (coenzyme Q, CoQ) and cyt *c* [73]. Reactions catalyzed by CI, CIII, and CIV result in the release of protons in the inner membrane space, thereby creating the proton gradient needed for ATP synthase activity. The correct function of the OxPhos system depends on the concerted action of several chaperones and other assembly factors that play essential roles in the formation, regulation, and stability of the five complexes and the mobile electron carriers, and nucleotide transporters [74]. Assembly factors of CI, CII, CIII, and CV have been classified as early-stage factors, acting in the structural assembly of individual subunits and sub-complexes, and late-stage accessory factors, called LYRM (leucine-tyrosine-arginine motif) proteins, controlling the incorporation and/or activation of last subunits and/or cofactors (i.e., Fe-S clusters). The human mitoproteome contains at least 12 LYRM proteins [75].

The OxPhos system is under a dual genetic control: 13 subunits are of mtDNA origin [24] and the remaining are encoded by the nuclear DNA (nDNA) [76]. MtDNA is a small circular genome [24] that encodes only 13 mitochondrial proteins, 22 mt-tRNAs, and 2 mt-rRNAs. Hence, the nuclear-encoded mitochondrial proteome requires sophisticated machinery for the transport into mitochondria [77,78,79]. Over the last years, a growing number of human proteins involved in mtDNA replication, and expression have been identified owing to the study of primary mitochondrial diseases. The coordination between the two genomes is crucial for mtDNA integrity, copy number regulation, and mitochondrial protein synthesis because mutations in nuclear genes encoding proteins for mtDNA replication and maintenance may affect its integrity and properties [80]. Dedicated reviews on these topics, including also the specific mechanisms regulating mtDNA replication [81], transcription [82], and translation [83,84] are available elsewhere.

Genetically, the mitochondrial diseases associated with the OxPhos system are split into two broad genetic categories: disorders due to mutations in the mtDNA, observing the rules of mitochondrial genetics; disorders due to mutations in the nDNA, transmitted as a Mendelian trait [6,85]. To date, mutations in both mitochondrial and nuclear genomes have been reported to cause mitochondrial disease manifesting with characteristic leukoencephalopathy and other clinical phenotypes either multisystemic or with single tissue involvement [86,87,88].

Since the first descriptions of mtDNA mutations [89,90,91], the number of mutations has been growing more and more until it counts over 1000 heteroplasmic rearrangements (large deletions/duplications) (http://mitobreak.portugene.com; accessed 25 July 2021), and over 500-point mutations possibly pathogenic among the 700 variants reported, which affect all mtDNA genes (https://www.mitomap.org; accessed 25 July 2021). A few major clinical phenotypes in adults have been recently reviewed [92]: LHON [91,93]; Neuropathy, ataxia, retinitis pigmentosa (NARP)/maternally inherited Leigh syndrome (MIILS) [94,95]; Maternally inherited nonsyndromic deafness, associated or not with aminoglycosides use [96]; Myoclonus, epilepsy, ragged-red-fibers syndrome (MERRF) [97,98]; Mitochondrial encephalopathy, lactic acidosis stroke-like syndrome (MELAS) [99,100]; Chronic progressive external ophthalmoplegia (CPEO) spectrum [89]; Kearns–Sayre syndrome (KSS) [101,102] and Pearson’s syndrome [103,104]. LHON and NARP/MILS are disorders that affect single OxPhos complex, complex I in LHON [105], and complex V in NARP/MILS [106], respectively. All these phenotypes are maternally inherited, displaying the hallmarks of mitochondrial diseases including variability of the phenotype, incomplete penetrance, and overlapping clinical features. The exception is represented by CPEO/KSS/Pearson associated with single mtDNA deletions, which are mostly sporadic [107,108]. 

Herein, we will provide some rapid information on structure, assembly, and disorders related to each of the OxPhos complexes. All the details of complexes assembly, including the factors, the interacting module/function, the associated clinical phenotypes, and the references have been adapted from [47,74,109,110].

### 4.1. NADH–Ubiquinone Oxidoreductase–Complex I 

NADH–Ubiquinone Oxidoreductase (Complex I, CI) couples the electron transfer of the two electrons derived from NADH oxidation to the ubiquinone with the translocation of four protons into the intermembrane space (IMS) [111,112,113]. Most of the molecular studies of mitochondrial diseases have focused on Complex I, which is the largest and most complicated among the respiratory complexes. Of 45 subunits, seven are encoded by the mtDNA (MT-ND1-6 and MT-ND4L), and the remaining, including the dual copy of the acyl-carrier protein NDUFAB1 [114], are encoded by nDNA [114,115]. Structurally, CI is an L-shaped complex that is composed of two domains: the hydrophilic head protruding into the matrix and the hydrophobic part within the inner mitochondrial membrane (IMM) [116]. Fourteen core subunits, conserved from bacteria to humans, perform catalytic activities [114,117,118]. Seven core subunits in the hydrophilic arm contain the redox-active centers: a non-covalently bound FMN and seven Fe–S clusters [119]. All the seven mtDNA-encoded CI subunits are in the hydrophobic arm and form the proton channels [115]. The remaining 30 subunits are ‘*supernumerary*’ but important for assembly and stability [120]. Most accessory subunits are only found in eukaryotic complex I. A notable exception is represented by subunits NDUFS4, NDUFS6, and NDUFA12 that are already present in complex I from α proteobacteria [121].

The complete mammalian CI structure has been elucidated [111,122] and determined by X-ray crystallography [117,123] and cryo-EM [118,124,125,126,127,128,129]. It is organized in six independent modules, N, Q, ND1/P_P-a_, ND2/P_P-b_, ND4/P_D,_ and ND5/P_D-b_, that, assisted by specific assembly factors, are incorporated in a specific order [130]. The overall L-shaped CI structure derives from the assembly of the N- and Q modules in the peripheral arm, and ND1, ND2, ND4, and ND5 modules in the P part of the membrane arm forming, at the hinge between the two arms, the channel of the CoQ binding site (Q-module) [119,120]. The N module, situated at the head of the hydrophilic part, contains the NADH-binding site and a flavomononucleotide (FMN) cofactor which oxidizes NADH to release two electrons [130]; the Q module for Q reduction, situated in the hydrophilic arm, contains eight Fe–S clusters where electrons flow to reach ubiquinone [130]. The N and Q modules form the peripheral arm containing the seven “core” subunits (NDUFV1, NDUFV2, NDUFS1, NDUFS2, NDUFS3, NDUFS7, and NDUFS8) whereas the 30 accessory subunits are necessary to stabilize the enzyme [131]. The P-module constitutes the membrane arm and is composed of the seven mtDNA-encoded proteins: ND1- ND4, ND4L, ND5, and ND6, involved in proton translocation [132]. Specific factors assisting the preassembly of the modules and the role of protein import machinery are summarized in Table 1.

A wide range of pathological phenotypes of the nervous system has been found to affect CI stability/activity both involving mitochondrial- and nuclear-encoded subunits [6]. Many pathological variants in the seven mtDNA encoded subunits, *MT-ND1*-6 and *ND*4L have been associated with a wide *spectrum* of syndromes with the age of onset occurring mostly during late childhood or early adulthood [178,179,180,181]. Mutations in three *MT-ND* genes are the main cause of Leber’s hereditary optic neuropathy (LHON) [OMIM 535 000], the most common mtDNA inherited disease [182]. LHON is one cause of bilateral acute or subacute, painless loss of central vision in young men (more than 80% of LHON patients are male, because of degeneration of retinal ganglion cell layers [183,184]. Important clues to understanding the pathogenesis of LHON, which is characterized by yet poorly understood genetic and environmental factors affecting the incomplete penetrance, have been obtained by analysis of mtDNA copy number and by proteomics approaches [185,186,187,188]. Mitochondrial DNA copy number is a key factor in differentiating LHON affected individuals from the unaffected mutation carriers [185,186,187,188]. A mitochondrial proteomic profile of 11778G>A mutant fibroblasts using 2-Dimensional Polyacrylamide Gel Electrophoresis (2-DE) and MS [189] disclosed that most of the mitochondrial proteins–including those involved in intermediary metabolic processes, nucleoid-related proteins, chaperones, *cristae* remodeling ones, and an antioxidant enzyme–were down-regulated, and some OxPhos subunits were altered [189]. The major bioenergetics consequences, particularly of *MT-ND4* and *MT-ND1* mutations, resulted in CI-dependent reduction of ATP synthesis and redox balance leading to increased ROS levels and decreased antioxidant enzyme activities [190,191,192]. 

The main pathological mutations found in structural CI subunits are summarized in Table 2.

Quantitative proteomics has revealed the importance of the 30 non-catalytically active supernumerary subunits of CI. Pathological variants causing CI deficiency have been described in NDUFAF1 [CIA30], ACAD9, and TMEM126B that together with ECSIT, COA1 and TMEM186, form the Mitochondrial Complex I Intermediate Assembly (MCIA) [172] important for the biogenesis of the ND2-module. NDUFAF3 (C3ORF60) and NDUFAF4 (C6ORF66) working together in the assembly of the Q-module, have been found mutated in different cases of infantile mitochondrial disease [150,151,228,229,230,231]. 

The gene *NDUFS4* (NADH dehydrogenase [ubiquinone] iron-sulfur protein 4, NM_002495.2), is a hotspot for pathogenic mutations. Inactivation of the *NDUFS4* gene is known to cause mostly, Leigh or Leigh-like syndrome [232,233,234,235,236,237,238,239,240], a rare disease with a prevalence of roughly 1:40.000 live births [241,242]. Unfortunately, the prognosis of *NDUFS4*-linked LS is poor. Loss of NDUFS4 affects complex I assembly and causes detrimental structural changes in assembled complex I [232,243]. Several pieces of evidence have suggested that NDUFS4 plays a role in the late stage of complex I assembly [233,235,244]. *NDUFS4* knock out mouse models [245,246], human and murine cell lines, and more recently induced pluripotent stem cells (iPSCs) from LS patients carrying mtDNA mutations in the *NDUFS4* [70] have been set up to explore strategies to counteract pathophysiological consequences of complex I deficiency. LS patient-derived neural cells have shown defective bioenergetics [247,248], decreased protein synthesis [249], impaired mitochondrial calcium homeostasis [248,250], and abnormal corticogenesis [251]. The presence of defective neurite outgrowth has been confirmed also in neural progenitor cells (NPCs) carrying mutations in the *NDUFS4* as well as in the *SURF1* (Surfeit locus protein 1, NM_003172.2) genes, another well-known cause of LS [252,253,254]. 

Structural subunits and specific factors assisting the assembly associated with human diseases are summarized in Table 1 and Table 2.

### 4.2. Succinate–Ubiquinone Oxidoreductase–Complex II

Succinate dehydrogenase (SDH, complex II, CII), a ~120 kDa integral membrane complex, participates in both the TCA cycle and the respiratory chain. CII transfers the electrons to CoQ and does not contribute to proton pumping across the mitochondrial membrane. All four subunits are encoded by the nuclear genome. The largest hydrophilic domain is a heterodimer composed of SDHA and SDHB that protrude toward the matrix and contain the redox-active groups’ flavin adenine dinucleotide (FAD(H2)) and three Fe–S clusters, respectively. The smaller hydrophobic domain is composed of SDHC and SDHD and contains two CoQ binding sites [255] providing reduction of ubiquinone to ubiquinol, the mobile electron carrier that links to CIII. Four specific chaperones [SDH assembly factor 1–4 (SDHAF1–4)] participate in the stabilization and incorporation of the prosthetic groups into each of the structural subunits SDHA, SDHB, and SDHC + SDHD [130,256]. In the late stage of assembly of CII, ACN9, similarly to LYRM-8 (also known as SDHAF1), is important for the formation and stabilization of CII throughout the insertion or retention of the Fe-S centers within the protein backbones and FMC1 (Formation of mitochondrial complex V assembly factor 1) [257]. 

CII defects are quite rare and represent less than 10% of OxPhos deficiency cases [258]. Different forms of encephalopathy and rare neuroendocrine tumors are the two main pathological manifestations that can originate from mutations in CII subunits or assembly factors. Mutations in SDHA, encoding the 70 kDa Flavoprotein subunit, have also been found in rare cases of Leigh syndrome [259,260,261,262,263,264]. Ultrarare association of bi-genomic variants in the SDHB and mitochondrial MT-CYB genes has been described in a patient with clinical and metabolic features of a ME-LAS-like syndrome [265].

The main pathological mutations found in CII subunits or assembly factors are summarized in Table 3.

### 4.3. Ubiquinol: Cytochrome C Oxidoreductase–Complex III

The ubiquinol: cytochrome c oxidoreductase (cytochrome bc1, complex III, CIII) constitutes the central part of the respiratory chain. CIII receives two electrons through reduced CoQ (CoQH2) and transfers them, one at a time, to cytochrome *c*, by cytochrome *b* (MT-CYB-human nomenclature), which contains two binding sites with CoQ and two heme *b* groups; UQCRFS1, the Rieske Fe-S protein; and CYC1, containing heme *c*. Each of the two ‘monomers’ is composed of 10 different subunits and associate as a symmetric dimer [279]. The complex assembly starts with the synthesis, membrane insertion, and hemylation of cytochrome b, mediated by UQCC1–3 in humans [280,281,282], followed by the sequential incorporation of the remaining subunits into a dimeric pre-CIII2 [282]. MZM1L (LYRM7), BCS1L, and tetratricopeptide repeat domain-containing protein 19 (TTC19) are the three assembly factors, known to be involved in the stabilization, incorporation, and metabolism of UQCRFS1 [283,284,285,286,287,288,289,290]. LYRM7 chaperone binds the Rieske protein before its incorporation as the last step of the biogenesis of the nascent CIII dimer (CIII2), acted by BCS1L [284,286,291].

The first mutations found in CIII were identified in MT-CYB, the only subunit encoded by mtDNA [292,293,294,295]. Most of these pathological variants were found in heteroplasmy and mainly associated with late-onset sporadic myopathy and exercise intolerance [292,293,294,295,296,297,298]. Other MT-CYB mutations were associated with histiocytoid cardiomyopathy [299], parkinsonism and MELAS overlap syndrome [293], or multisystem disorders [300,301,302,303]. 

Among the cases of CIII deficiency of nuclear origin are mutations in assembly factors [304] and the most common are nonsense and missense mutations in TTC19 [305], LYRM7 [306], and BCS1L [307], which cause defective CIII assembly/stability and decreased ubiquinol:cyt *c* oxidoreductase activity. Interestingly, a shuttle of electrons from NADH and/or ubiquinol to CIII, pyocyanin, has been used to efficiently recover mitochondrial function thus ameliorating bioenergetic efficiency in fibroblasts derived from patients’ dysfunction due to TTC19, BCS1L, and LYRM7 [291]. 

The main pathological mutations found in CIII subunits or assembly factors are summarized in Table 4.

### 4.4. Cytochrome C Oxidase–Complex IV 

Cytochrome *c* oxidase (COX, complex IV, CIV) is the terminal complex of the ETC. The enzyme transfers electrons from cytochrome *c* to molecular oxygen. In humans, it is composed of 14 subunits, with the NDUFA4, the most recently discovered subunit initially attributed to CI [331,332], found to be incorporated in the structure of monomeric human CIV [333]. Only two, MT-CO1 and MT-CO2, are catalytical subunits. MTCO1 contains three prosthetic groups: cytochrome *a*3 and CuB, which form the bi-nuclear center that binds oxygen, and cytochrome *a*. MT-CO2 incorporates the CuA center [334]. MT-CO3 is necessary to provide additional stability to the enzyme while it undergoes turnover [335]. Subunits such as COX4, 5A, 5B, 6A, 6B, 6C, 7A, 7B, 7C, 8A are believed to play a role in stabilizing the structure of the complex. The cytochrome *c* oxidase complex is unique among the ETC complexes to have tissue, developmental and species-specific isoforms for COX subunits 4, 6A, 6B, 7A, 7B, and 8A [336,337].

CIV assembly grows with a modular process through the incorporation of modules formed by different subunits and defined by each of the mtDNA-encoded core subunits [130,338,339]. Any subunit of complex IV could carry mutations and rise a mitochondriopathy [337,340,341,342]. Mutations in the MT-CO1, MT-CO2, and MT-CO3 are causative of COX deficiency and mitochondrial disease with an extreme clinical heterogeneity (Table 5).

Pathological variants in ‘*supernumerary*’ COX subunits have been reported in tissue and development-specific isoforms [336]. Among the assembly factors, the most representative is *SURF1*, the functional absence of which causes LS [252,253,276] or even Charcot–Marie–Tooth disease [367]. The elucidation of the pathogenetic mechanism has received an impulse recently [70]. Mutations in *COX*10, which catalyzes the farnesylation of a vinyl group of heme *b*, cause LS and other forms of the fatal early-onset neurological syndrome [368,369,370]. Mutations in *COX*15, which catalyzes the subsequent step of heme synthesis, cause variable clinical presentations [371,372,373]. Copper delivery to the active sites of *MT-CO*1 and *MT-CO*2 involves factors essential for COX activity [130,374]. *SCO*1, *SCO*2, and *COA*6 have been found mutated in patients showing CIV deficiency and fatal outcomes [338,368,375,376,377,378,379,380,381,382,383,384,385,386]. Among complex IV proteins, COX6B1 assists CIV assembly, working as a linking subunit at the dimeric interface of CIV [387].

The specific functions of the remaining proteins (all associated with human diseases, see Table 6) are known only in part and require additional studies.

### 4.5. ATP Synthase–Complex V

ATP synthase (Complex V, CV) is the enzyme that catalyzes the synthesis of ATP required as an energy source for various cellular processes from ADP and phosphate utilizing the proton-motive force generated through electron transfer. ATP synthase F_1_F_O_ consists of two functional domains: the hydrophilic domain F_1_ facing the matrix which serves for the production of ATP and the F_O_ domain facing the membrane which serves to translocate protons [433,434]. The proton translocation leads to the rotational movement of the c ring in the F_O_ domain which is connected to the catalytic subunit F1 by the peripheral stalk (PS). The human CV is composed of 29 proteins of 18 kinds, including the Inhibitory factor 1, IF_1_, in which only F_O_-ATP_6_ and ATP_8_ are mtDNA encoded [435]. The complete structure of the dimeric and monomeric mammalian mitochondrial F_1_Fo-ATP synthase has been just recently resolved by Cryo-EM [436,437].

The assembly pathway of human CV is also modular [433,435,438,439] since three subcomplexes, F*1* module, c-ring, and PS are formed individually and then associate together. The assembly starts from the three alpha and three beta subunits that make up the F1 domain to which the other subunits subsequently bind. The eight units of the c-ring assemble inside the IMM. When these two sub-complexes join, the PS subunits also bind, followed by the membrane domain’s remaining subunits, which include MT-ATP6 and MT-ATP8 [130]. To date, only three assembly factors are known, including ATPAF1 and ATPAF2, that binds and stabilizes subunit beta [440] and subunit alpha [441], respectively.

Pathogenic mutations have been reported both in mtDNA and nDNA encoded ATP synthase subunits. The coding sequences of two Fo subunits are overlapping in the human mtDNA and pathological variants in both are the cause of sporadic and maternally inherited mitochondrial disease (Table 7).

Mutations in MT-ATP6 have been identified in neuropathy, ataxia, and retinitis pigmentosa syndrome (NARP) [94] and maternally inherited Leigh syndrome (MILS) [442,466]. NARP is a slowly progressive form that manifests in adulthood, while MILS is an early onset, highly disabling, often fatal disease. In many cases, NARP and MILS are associated with the 8993 T > C or T > G mutation [443,467,468]. The T > G transversion usually presents with a more severe form that correlates with the degree of heteroplasmy of the mutation in post-mitotic tissues [95,468]. Until now, only three of the sixteen nucleus-encoded CV subunits and three assembly factors (e.g., ATPAF2; ATP12 and TMEM70) have been associated with mitochondrial disease (see Table 7). 

### 4.6. Respiratory Supercomplexes

OxPhos complexes associate with each other resulting in the formation of higher-order structures which have been called supercomplexes (SC). Complexes IV and V can form dimers and oligomers [469,470,471] and based on the size and composition of the subunits, the main SCs that have been recognized have the following stoichiometries: III_2_IV_1_, I_1_III_2_, I_1_III_2_IV_1_, and I_2_III_2_IV_1-2_. In particular, the association of complexes I, III2 and IV, SC I_1_III_2_IV_1_, considered as a functional unit capable of transferring electrons from NADH to O_2_, is defined as the ‘respirasome’ [472] whereas the supercomplex I_2_III_2_IV_2_ has been named as ‘respiratory megacomplex’ [129]. High-resolution Cryo-EM structures of the respirasome of several mammalian species, including humans, have been recently resolved [129,473,474,475,476]. The respirasome organization was supposed to be functionally advantageous making electron transfer from CI to CIV through CIII_2_ more efficient and decreasing the formation of deleterious ROS [477,478,479,480]. It has been suggested that the functional unit of OxPhos is composed of the dimer of ATP synthase flanked by the adenine nucleotide and the phosphate transporters, located at the apices of cristae and the CI-CIII_2_-CIV supercomplexes organized along the *cristae* membrane to perform the electron transfer and proton translocation [481].

The fact that the biogenesis of CIII_2_ and CIV occurs independently but the CI assembly does not can be an explanation for the reason why defects in CIII_2_ and CIV may result in secondary effects on CI assembly. When the defect is originated from mutations in CI components, the manifestation is almost always an isolated CI deficiency [209,482]. High-throughput proteomics techniques have recently been applied in human cybrids holo-CIII_2_-deficient, demonstrating the loss of SCs containing CIII_2_ and CI when the CIII_2_ is not fully assembled. In this model, the combination of null CIII and markedly reduced CI enzymatic activity, confirmed the well-established connection between CIII_2_ deficiency and hampered assembly process in CI [483].

In astrocytes, most of CI is free, resulting in poor mitochondrial respiration but high ROS production; while, in neurons, CI is mostly embedded into supercomplexes, thus resulting in high mitochondrial respiration and low ROS production [66]. Notably, crest-shaping proteins, as well as the proteins of mitochondrial contact sites and the cristae organization system complex (MICOS) are essential for the assembly and functionality of the OxPhos system [484]. Understanding the structure and assembly of SCs is very crucial to explain those cases of combined respiratory chain deficiency.

For expert reviews on the issue of the relationship between crest dynamics and bioenergetics, refer to [485,486]. For a detailed review of the formation and function of SCs, see [487].

## 5. Conclusions

An integrative approach that combines multi-omics data could represent a strategic way to solve, at least in part, the complexity of mitochondrial diseases and mitochondrial medicine highlighting the interrelationships of the involved OxPhos complexes and their functions, and the knowledge about genotype-phenotype correlation. However, the science behind combined omic approaches, will need the integration of data from genomics, transcriptomics, proteomics, and metabolomics, to include also the novel approaches looking to the epiproteome, the set of all post-translational modifications made to proteins comprising an organelle, a cell, or an organism, that provide the link between metabolism, mitochondrial proteome, and the two cellular genomes. The recent application of CRISPR/Cas9 technology to patient-specific iPSCs, to generate neural cultures and cerebral organoids is providing patient-specific cellular and tissue models that allow the investigation of the defects of neuronal morphogenesis caused by specific mutations (Figure 1). Thus, in the multi-omic era, the opportunity to understand the cause of each mitochondrial disease becomes ever more tangible.

## Figures and Tables

**Table 1:** Complex I assembly factors with interacting module/function, associated clinical phenotypes, and references. Adapted from [47,74,110,133].

Assembly Factors	CI Interacting Module/Function	Associated Clinical Phenotypes	References
ACAD9	ND2/PP-b module Component of MCIA complex, necessary for insertion of ND2	Cardiorespiratory depression, hypertrophic cardiomyopathy, encephalopathy, and severe lactic acidosis	[134,135]
ECSIT	ND2/PP-b module Component of MCIA complex, necessary for insertion of ND2	-	[136]
FOXRED1	ND4/PD module	Leigh syndrome, congenital lactic acidosis, athetoid movements of the limbs in early childhood, hypotonia and cerebellar atrophy, mitochondrial respiratory CI deficiency associated with Leigh syndrome, encephalocardiomyopathy, or ataxia	[137,138,139]
ATP5SL/DMAC2	ND4/PD module	-	[140]
TMEM70	ND4/PD module	Neonatal mitochondrial encephalocardiomyopathy, mitochondrial CV deficiency, nuclear type 2, occasionally facial dysmorphisms and CI deficiency	[141,142,143,144,145,146]
NDUFAF1	N module, ND1 Component of MCIA complex, necessary for insertion of ND2	Hypertrophic cardiomyopathy, developmental delay, lactic acidosis, hypotonia, and Wolff–Parkinson–White syndrome	[147,148]
NDUFAF2	N module. Stabilization of pre-CI or 830 kDa subcomplex	Ataxia, lethargy, nystagmus, hypotonia, optic atrophy, and episodic respiratory, insufficiency, generic encephalopathic syndromes, or Leigh syndrome	[149]
NDUFAF3/C3ORF60	Q module	Macrocephaly, weak cry, no eye contact, wide anterior fontanel and axial hypotonia	[150]
NDUFAF4/C6ORF66	Q module	Severe encephalopathy and antenatal Cardiomyopathy	[151]
NDUFAF5/C20ORF7	Not known. Catalyze hydroxylation of NDUFS7 and dimethylation of NDUFS2 of the Q module	Facial dysmorphism, progressive lactic acidosis and neurological defects, severe early-onset encephalopathy	[152,153]
NDUFAF6	Not known. Maintain a normal level of mt-ND1 subunit	Focal seizures, decreased movement and strength, ataxia, lactic acidosis, and Leigh syndrome	[29,154,155,156,157,158]
NDUFAF7	Not known. Catalyze dimethylation of NDUFS2 of the Q module	-	[159,160]
NDUFAF8/C17ORF89	Not known. Stabilization of NDUFAF5	Leigh syndrome	[161]
NUBPL	Supposed to interact with the developing N module and possibly Q module. Insertion of iron-sulfur clusters in N and Q module subunits	Infantile onset hepatopathy, renal tubular acidosis, developmental delay, short stature, leukoencephalopathy, myopathy, nystagmus, and ataxia	[162,163,164]
TIMMDC1/C3ORF1	ND1/PP-a Insertion of ND1	Infantile onset hypotonia, failure to thrive, delayed or minimal psychomotor development, sensorineural deafness, dysmetria, dyskinetic movements, peripheral neuropathy, nystagmus, and Leigh syndrome	[140,165,166]
TMEM126A	ND4 module Component of MCIA complex, necessary for building the intermediate ND2 module	Autosomal recessive optic atrophy	[167,168,169,170,171]
TMEM126B	ND2/PP-b module Component of MCIA complex, necessary for building the intermediate ND2 module	Exercise intolerance, muscle weakness, myalgia, early-onset renal tubular acidosis, and hypertrophic cardiomyopathy	[172,173,174]
TMEM186	ND2/PP-b module- Interact strongly with newly synthesized ND3	-	[175]
DMAC1/TMEM261	ND5/PD-b	-	[120]
COA1/MITRAC15	ND2/PP-b module	-	[175]
COA7	-	Autosomal recessive spinocerebellar ataxia with axonal neuropathy type 3	[176]
LYRM-2	NADH-Dehydrogenase module Maturation of N-module	-	[177]

**Table 2:** Complex II subunits with location, associated clinical phenotypes, and references. Adapted from [47,74,110,133].

Subunits	Location	Associated Clinical Phenotypes	References
MTND1	ND1-module	Leber optic atrophy, MELAS syndrome, dystonia, spasticity, and myopathy	[193,194,195]
MTND2	ND2-module	Leber optic atrophy	[196]
MTND3	ND2-module	Infantile encephalopathy and Leigh syndrome	[197]
MTND4	ND4-module	Leber optic atrophy and MELAS syndrome	[198,199]
MTND4L	ND2-module	Leber optic atrophy	[200]
MTND5	ND5-module	Leber optic atrophy and MELAS syndrome	[201,202]
MTND6	ND2-module	Leber optic atrophy and MELAS syndrome	[201,203]
NDUFV1	N-module	Severe encephalopathy and neurologic abnormalities	[204,205]
NDUFV2	N-module	Hypertrophic cardiomyopathy, truncal hypotonia, and encephalopathy	[206]
NDUFV3	N-module	Complex I deficiency	-
NDUFS1	N-module	Growth retardation, axial hypotonia, hepatomegaly, dystonia, and persistent hyperlactatemia	[205]
NDUFS2	Q-module	Neonatal lactic acidosis and hypertrophic cardiomyopathy	[207]
NDUFS3	Q-module	Leigh syndrome, severe axial dystonia with oral and pharyngeal motor dysfunction, dysphagia and a tetraparetic syndrome	[208]
NDUFS4	Q-module	Muscular hypotonia, absence of visual and auditive attention, and cardiac defects	[209]
NDUFS6	Q-module	Fatal infantile lactic acidosis, neonatal myopathy, encephalopathy, and lactic acidosis	[210,211]
NDUFS7	Q-module	Leigh syndrome, feeding problems, dysarthria, and ataxia	[212]
NDUFS8	Q-module	Leigh syndrome, poor feeding, and episodes of apnea and cyanosis	[213]
NDUFA11	ND2-module	Fatal infantile metabolic acidosis, brain atrophy, no motor development and hypertrophic cardiomyopathy	[214]
NDUFA1	ND1-module	Leigh syndrome, hypotonia, nystagmus, generalized choreoathetosis, and decreased reflexes	[215]
NDUFA2	N-module	Leigh syndrome, hypertrophic cardiomyopathy, and developmental delay	[216]
NDUFA3	ND1-module	-	-
NDUFA5	Q-module	-	-
NDUFA6/LYRM-6	LYR protein	Auditory and optic neuropathy, mitochondrial-related infantile death, brain disorder, leukoencephalopathy	[217]
NDUFA7	N-module	-	-
NDUFA8	IMS protein (ND1-module)	Intrauterine growth retardation, respiratory insufficiency, lactic acidosis and hypoglycemia	[178]
NDUFA9	Q-module	Severe neonatal hypotonia, dysmorphic features, epilepsy, and signs of brainstem involvement	[218]
NDUFA10	ND2-module	Leigh syndrome	-
NDUFA11	ND2-module	Encephalocardiomyopathy and fatal infantile lactic acidemia, neuromuscular disorder	-
NDUFA12	N-module	Respiratory and metabolic acidosis, hearing loss, apneas, and retinitis pigmentosa	[219]
NDUFA13	ND1-module	Leigh syndrome, progressive loss of motor abilities, scoliosis, and dystonia	[220]
NDUFB1	ND4-module	-	-
NDUFB2	ND5-module	-	-
NDUFB3	ND5-module	Delayed development, hypotonia, poor eye contact, abnormal eye movements, poor feeding, encephalopathy, and hearing loss	[221]
NDUFB4	ND4-module	-	-
NDUFB5	ND4-module	-	-
NDUFB6	ND5-module	-	-
NDUFB7	ND5-module	-	-
NDUFB8	ND5-module	Encephalopathy, myopathy, hypotonia, developmental delay, and lactic acidosis, mitochondrial Complex I Deficiency in Individuals with Leigh-like Encephalomyopathy	[222]
NDUFB9/LYRM-3	LYR protein	Leigh syndrome, respiratory failure, seizures, hypotonia, cardiac hypertrophy, failureto thrive and severely delayed psychomotor development	[221]
NDUFB10	IMS protein(ND4 module)	Progressive hypotonia associated with increased serum lactate	[223]
NDUFB11	ND4-module	Lethal complex I deficiency, X-linked microphthalmia with linear skin defects (MLS) syndrome	[224,225,226]
NDUFC1	ND2-module	-	-
NDUFC2	ND2-module	X-linked microphthalmia with linear skin defects (MLS) syndrome, cardiomyopathy and other congenital anomalies	[227]
NDUFS5	IMS protein (ND2 module)	-	-

**Table 3:** Complex II subunits and assembly factors with function, associated clinical phenotypes, and references. Adapted from [74,133].

Subunits	Function	Associated Clinical Phenotypes	References
SDHA	CII subunit	Leigh syndrome, neonatal dilated cardiomyopathy, catecholamine-secreting extra-adrenal paraganglioma	[259,260,261,262,263,264,265,266,267]
SDHB	CII subunit	Paraganglioma, pheochromocytoma, gastrointestinal stromal tumors	[268,269]
SDHC	CII subunit	Paraganglioma, gastric stromal sarcoma	[270,271]
SDHD	CII subunit	Paraganglioma, pheochromocytoma, gastric stromal sarcoma	[271,272]
**Assembly Factors**			
SDHAF1/LYRM-8	Insert Fe/S clusters into mature SDHB	Leukoencephalopathy, spastic quadriplegia, psychomotor regression	[257]
SDHAF2	Insert FAD cofactor into apo-protein SDHA	Paraganglioma and pheochromocytomas	[270,272,273,274,275,276]
SDHAF3/NDUFV1/LYRM-10	Maintain SHDB stability	Familial and sporadic pheochromocytomas and paraganglioma	[277]
SDHAF4	Protect the subunit from auto-oxidation and facilitates the assembly with SDHB	Vagal paragangliomas	[278]

**Table 4:** Complex III subunits and assembly factors with function, associated clinical phenotypes, and references. Adapted from [74,133].

Subunits	Function	Associated Clinical Phenotypes	References
UQCRC1	CIII subunit	Parkinsonism with polyneuropathy	[308]
UQCRC2	CIII subunit	Hypoglycemia, lactic acidosis, ketosis, and hyperammonemia	[309]
MT-CYB	CIII subunit	Leber optic atrophy, exercise intolerance, encephalomyopathy, cardiomyopathy, and multisystemic disorder, histiocytosis cardiomyopathy, parkinsonism, and MELAS overlap syndrome	[293,294,299,300,310,311]
CYC1	CIII subunit	Neurologic deterioration, insulin-responsive hyperglycemia, ketoacidosis with increased serum lactate, liver failure, and hyperammonemia	[312]
UQCRFS1	CIII subunit	Cardiomyopathy and alopecia totalis	[313]
UQCRH	CIII subunit	-	-
UQCRB	CIII subunit	Gastroenteritis, liver enlargement, hypoglycemia, and metabolic acidosis but normal psychomotor development at age 4, hepatopathy	[314]
UQCRQ	CIII subunit	Severe neurologic phenotype, early-onset severe encephalopathy	[315]
UQCR10	CIII subunit	-	-
UQCR11	CIII subunit	-	-
**Assembly Factors**			
UQCC1	Cytochrome *b* assembly factor	-	-
UQCC2	Cytochrome *b* assembly factor	Intrauterine growth retardation, neonatal lactic acidosis and renal tubular dysfunction	[281,316]
UQCC3	Cytochrome *b* assembly factor	Lactic acidosis, hypoglycemia, hypotonia, and delayed development	[282]
VPS53	Heme lyase (Cytochrome c1)	Complicated hereditary spastic paraparesis	[317]
BCS1L	AAA-ATPase involved in Rieske protein incorporation. Stabilization, incorporation, and metabolism of UQCRFS1	GRACILE Syndrome, Bjornstad Syndrome, myopathy, encephalopathy, proximal tubulopathy, and liver failure	[26,288,304,318,319,320,321,322,323]
MZM1L/LYRM-7	Matrix protein involved in Rieske protein incorporation. Stabilization, incorporation, and metabolism of UQCRFS1	Neurological decompensation and regression, leukoencephalopathy and liver failure, infantile CIII deficiency associated with cavitating leukoencephalopathy metabolic decompensation	[306,324,325,326]
TTC19	Rieske protein metabolism Stabilization, incorporation, and metabolism of UQCRFS1	Progressive encephalopathy, ataxia, spastic paraparesis, and psychiatric phenotype	[305,327,328,329,330]

**Table 5:** Complex IV subunits with associated clinical phenotypes and references. Adapted from [74,133].

Subunits	Associated Clinical Phenotypes	References
MTCO1	MELAS syndrome, myopathy, myoglobinuria, motor neuron disease, exercise intolerance, epilepsy, multisystem disorders, deafness, LHON, or mitochondrial sensorineural hearing loss	[343,344,345,346,347]
MTCO2	Encephalomyopathy, LHON, myopathy, hypertrophic cardiomyopathy	[348,349,350,351]
MTCO3	MIDD, LHON, myopathy, Leigh disease, myoglobinuria, sporadic bilateral optic neuropathy, rhabdomyolysis, encephalopathy	[352,353,354,355,356,357]
COX4I1	Short stature, poor weight gain, mild dysmorphic features, Fanconi anemia, Leigh-like syndrome	[358,359]
COX4I2	Exocrine pancreatic insufficiency, dyserythropoietic anemia, calvarial hyperostosis	[360]
COX5A	Early-onset pulmonary arterial hypertension, lactic acidemia, failure to thrive	[361]
COX6A1	Charcot–Marie–Tooth disease	[362]
COX6A2	Muscle weakness and hypotonia, cardiomyopathy	[363]
COX6B1	Severe infantile encephalomyopathy	[341,342]
COX7A1	Failure to thrive, encephalopathy, hypotonia	[364]
COX7B	Microphthalmia with linear skin lesions	[365]
COX8A	Leigh-like syndrome presenting with leukodystrophy and severe epilepsy	[366]
NDUFA4	Leigh syndrome	[331]

**Table 6:** Complex IV assembly factors with function, associated clinical phenotypes, and references. Adapted from [74,133].

Assembly Factors	Function	Associated Clinical Phenotypes	References
	**RNA Stability and Translation**		
TACO1	Translational activator of mitochondria encoded MTCO1	Leigh syndrome	[388,389]
LRPPRC	Mitochondrial mRNA stability	French Canadian type of Leigh syndrome	[390]
FASTKD2	Involved in post-transcriptional RNA maturation, ribosome biogenesis and translation	Brain atrophy, epilepsy, delayed psychomotor development, bilateral optic atrophy, spastic hemiparesis, cardiomyopathy	[391,392,393]
	**Heme *a* Biosynthesis and Insertion**		
COX10	Heme a synthesis (conversion of heme b into heme o)	Leigh syndrome, encephalopathy, cardiomyopathy, sensorineural deafness, and metabolic acidosis	[369,370,394,395]
COX15	Heme a synthesis (conversion of heme o into heme a)	Leigh syndrome, encephalopathy, cardiomyopathy, sensorineural deafness, and metabolic acidosis	[369,371,373,396,397]
SURF1	Involved in the insertion or stabilization of heme a3	Leigh syndrome, Charcot–Marie–Tooth disease	[252,253,276,367,398]
	**Copper Metabolism and Insertion**		
COA5/C2ORF64	Involved in the unknown step of CIV biogenesis	Fatal infantile cardioencephalomyopathy	[399]
COA6/C1ORF31	Copper homeostasis and transport to CIV	Fatal infantile cardioencephalopathy	[385,386,400]
SCO1	Incorporation of copper atoms (biogenesis of CuA center)	Cardioencephalomyopathy, Leigh syndrome-like symptoms, spinal muscular atrophy-like presentations, Charcot–Marie–Tooth disease type 4, CIV deficiency, neonatal hepatopathy, encephalopathy with hepatopathy and cardiomyopathy, pure encephalopathy, metabolic syndrome with exclusively fatal lactic acidosis	[375,381,383,395,401,402]
SCO2	Incorporation of copper atoms (biogenesis of CuA center)	Cardioencephalomyopathy, Leigh syndrome-like symptoms, spinal muscular atrophy-like presentations, Charcot–Marie–Tooth disease type 4, CIV deficiency, cardiac hypertrophy	[377,378,379,380,381]
COX11	Copper chaperone	Coloboma, Ocular, With or Without Hearing Impairment, Cleft Lip/Palate, And/Or Mental Retardation and Spinal Muscular Atrophy, Distal, X-Linked 3	[403]
COX16	MTCO2 maturation	-	[404,405]
COX17	Copper transfer	-	[406]
COX19	Stabilization of COX11	-	[407,408]
COX20	Stabilization of MT-CO2	Cerebellar ataxia	[409,410,411]
	**Assembly**		
COA3/MITRAC12	Required for MTCO1 stability and assembly, involved in translational regulation of MTCO1 and prevention of MTCO1 aggregation before assembly	Mild phenotype, exercise intolerance, peripheral neuropathy, obesity, and short stature	[412,413,414,415]
COA7	Unknown	Ataxia and peripheral neuropathy, cognitive impairments, leukodystrophy	[176,416]
COX14/C12ORF62	MTCO1 stability and assembly; avoids MTCO1 aggregation	Severe lactic acidosis and dysmorphic features	[417]
CMC1	Stabilizes the interaction between MTCO1, COX14, and COA3		[418]
COX20/FAM36A	MTCO2 chaperone for copper metalation	Growth delay, hypotonia, cerebellar ataxia	[410,411,419]
PET100	Stabilizes MT-CO2 module	Early-onset psychomotor delay, seizures, hypotonia, Leigh syndrome, CIV deficiency, and fatal infantile lactic acidosis	[420,421,422]
PET117	Assembly factor: possible role in Cox15 oligomerization and function, stabilizes MT-CO2 module	Neurodevelopmental regression and bulbar lesions	[423,424,425]
MR-1S	Interacts with PET117 and PET100,	-	[339]
APOPT1/COA8	intermediates assembly steps Putative role in CIV protection from ROS damage, enhances CIV biogenesis	Leukodystrophy, neurological signs	[426,427,428]
COX18	Promotes the translocation of MTCO2 globular domain through the IMM	Isolated COX deficiency in infancy	[429,430,431]
COX19	Stabilization of COX11	Isolated COX deficiency in infancy	[407,408,431]
COA-X	Putative assembly factor	-	[432]
HIGD2A	Promotes incorporation of MT-CO3 module	-	-

**Table 7:** Complex V subunits and assembly factors with function, associated clinical phenotypes, and references. Adapted from [74,133].

Subunits	Location	Associated Clincial Phenotypes	References
MT-ATP6	Fo domain	Mitochondrial CV deficiency Neuropathy, Ataxia and Retinitis Pigmentosa (NARP) syndrome Leigh syndrome Adult-onset ataxia and polyneuropathy Bilateral striatal necrosis Motor neuron syndrome Mitochondrial myopathy, lactic acidosis, and sideroblastic anemia	[94,95,442,443,444,445,446,447,448,449,450,451,452,453,454,455,456,457]
MT-ATP8	Fo domain	Mitochondrial CV deficiency Valproate-induced reversible brain atrophy Hypertrophic cardiomyopathy	[458,459]
MT-ATP6/8 overlap region	Fo domain	Mitochondrial CV deficiency Infantile hypertrophic cardiomyopathy	[457]
ATP5F1A	F1 domain	Mitochondrial CV deficiency Combined OXPHOS deficiency Fatal infantile encephalopathy	[460,461]
ATP5F1D	F1 domain	Mitochondrial CV deficiency Metabolic decompensation with lactic acidosis, hypoglycemia, hyperammonemia, and 3-methylglutaconic aciduria, encephalopathy	[462]
ATP5F1E	F1 domain	Mitochondrial CV deficiency Neonatal-onset lactic acidosis, 3-methylglutaconic aciduria, mild mental retardation, hypertrophic cardiomyopathy, and peripheral neuropathy	[463]
**Assembly Factors**			
ATPAF1	Binds and stabilIzes subunit beta of F1 Domain	Asthma in children	[464]
ATPAF2	Binds and stabilizes subunit alpha of F1 domain	Degenerative encephalopathy, elevated lactate levels, developmental delay	[465]
TMEM70	Unknown	Neonatal mitochondrial encephalocardiomyopathy Mitochondrial CV deficiency, nuclear type 2 Occasionally facial dysmorphisms CI deficiency	[141,142,143,144,145,146]

## Data Availability

Not applicable.
